# Nonsyndromic Primary Diffuse Leptomeningeal Melanomatosis in a Child

**DOI:** 10.22037/ijcn.v15i2.20159

**Published:** 2021

**Authors:** Salim TAVANA RAD, Farah ASHRAFZADEH, Hassan GOLMAKANI, Babak GANJEIFAR

**Affiliations:** 1 Department of Neurological Surgery, Ghaem Hospital, Faculty of Medicine, Mashhad University of Medical Sciences, Mashhad, Iran; 2 Department of Pediatrics, Ghaem Hospital, Faculty of Medicine, Mashhad University of Medical Sciences, Mashhad, Iran

**Keywords:** Spine, Central nervous system, Melanoma, Child

## Abstract

Introduction: In this study, we present a case of primary diffuse leptomeningeal melanomatosis (PDLM), without neurocutaneous melanosis syndrome.

A female patient (age: 14 years) presented with headache, nausea, vomiting, vertigo, diplopia, and lower limb weakness. The magnetic resonance imaging (MRI) showed leptomeningeal isointensity on T1- and T2-weighted images and hyperintensity on fluid attenuation inversion recovery (FLAIR) sequences. Definitive histological examination showed a densely cellular tumor, characterized by irregular clusters of large pleomorphic cells and melanin in tumor cells. Adjuvant therapy was refused by the parents, and the patient died within six months.

Primary diffuse leptomeningeal melanomatosis is recognized as an uncommon and malignant melanoma affecting the central nervous system. In case comorbidities are not diagnosed in patients with unusual symptoms of meningitis, diagnostic methods such as cerebrospinal fluid analysis and central nervous system biopsy can be helpful in identifying other underlying conditions.

## Introduction

Primary and malignant melanoma of the central nervous system (CNS) is an uncommon phenomenon, accounting for nearly 1% of all reported cases of melanoma. According to statistics, primary melanoma of the CNS occurs in 0.005 per 100 000 people (1). These tumors are mostly detected in adults and commonly affect individuals in their 40s (2); however, the tumor is uncommon during childhood (3).

Primary diffuse leptomeningeal melanomatosis (PDLM) is recognized as a rare type of primary malignant melanoma of the CNS. This condition in children can be associated with neurocutaneous melanocytosis, which is an uncommon congenital condition, identified by large (or various) melanocytic nevi in the skin and melanocytic leptomeningeal tumors (either benign or malignant) (3). In this paper, a case of PDLM, without the associated neurocutaneous melanocytosis, is presented.

## Case Report

A female patient (age: 14 years) with headache, nausea, vomiting, vertigo, diplopia, and lower limb weakness was admitted to the pediatric ward of Ghaem Hospital, Mashhad, Iran in May 2014. Neurologic examination showed flaccid weakness of the lower limbs (4/5). Pathologic reflexes were not detected, and sphincters were intact. On fundoscopy, the optic discs were normal.

The magnetic resonance imaging findings showed leptomeningeal isointensity on T1- and T2-weighted images and hyperintensity on fluid attenuation inversion recovery (FLAIR) sequences. These findings showed improvement after the intravenous administration of gadolinium, which was suggestive of carcinomatosis or microbial meningitis (Fig-1A, B and Fig2-A, B). 

**Fig-1 A:**
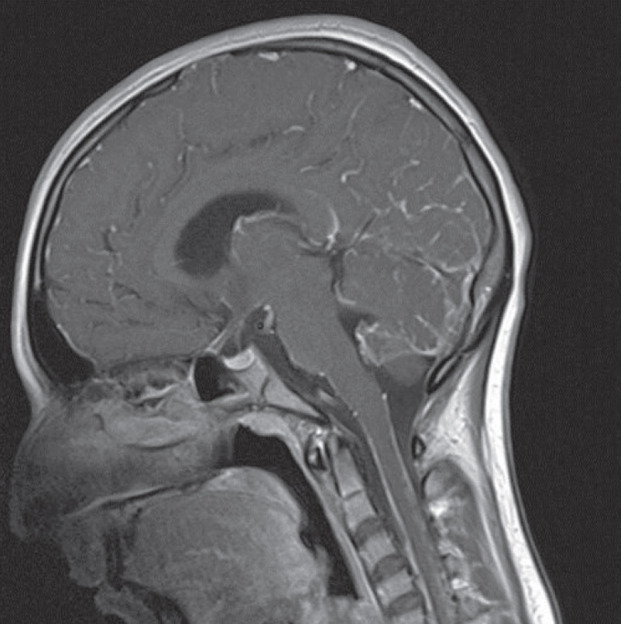
Brain magnetic resonance imaging (MRI) revealed diffuse enhancement after intravenous gadolinium injection

**Fig-1 B:**
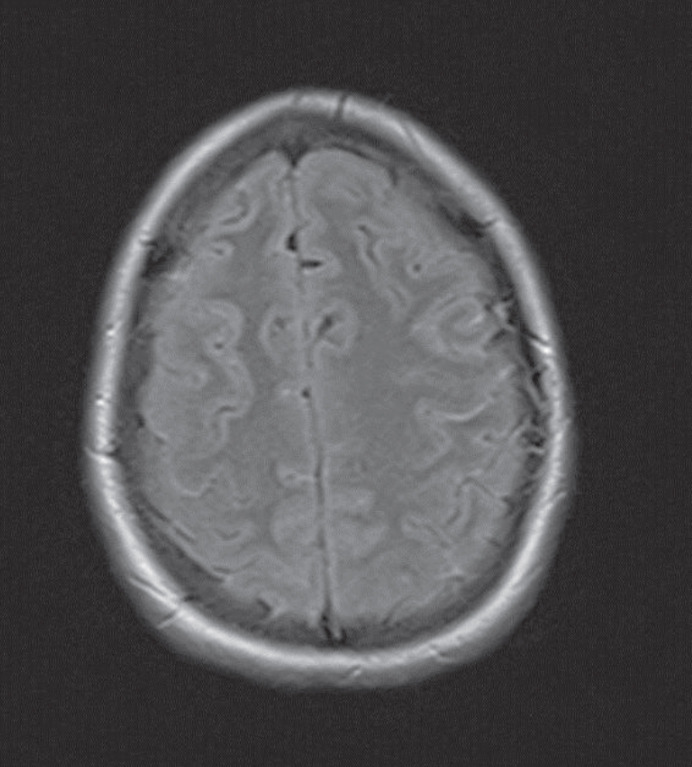
Brain magnetic resonance imaging (MRI) revealed diffuse enhancement after intravenous gadolinium and hyperintensity on fluid-attenuated inversion recovery sequence

**Fig-2 A:**
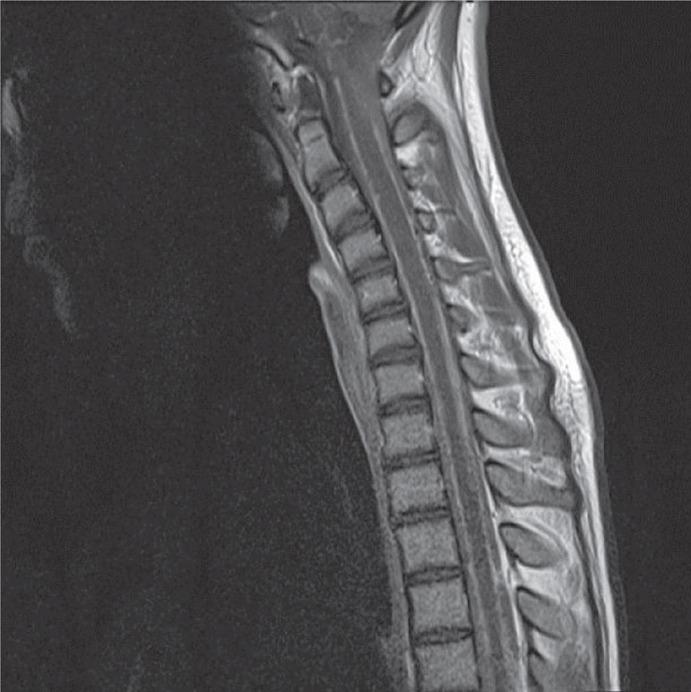
Spinal magnetic resonance imaging (MRI) on T1-weihgted demonstrated diffuse enhancement after intravenous gadolinium injection in cervical

**Fig-3 B:**
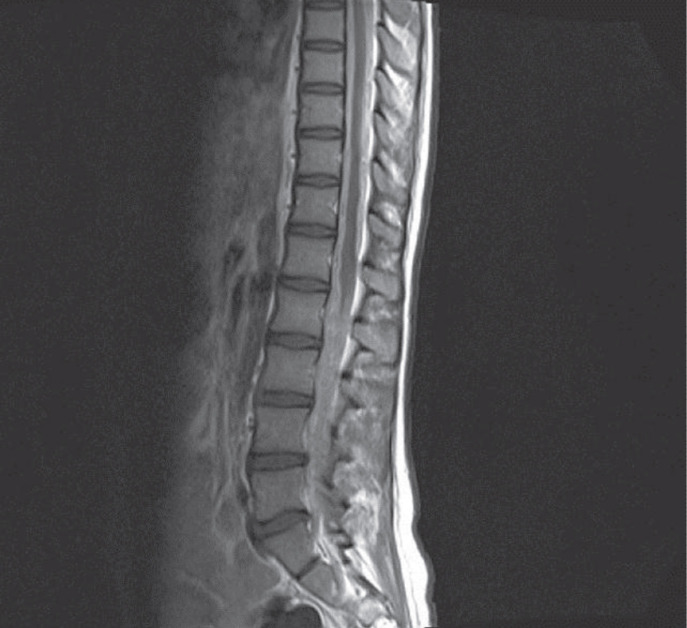
Spinal magnetic resonance imaging (MRI) on T1-weihgted demonstrated diffuse enhancement after intravenous gadolinium injection in cervical lumbosacral regions

Multiple lumbar punctures were found to be dry. Empirical treatment for bacterial meningitis was initiated. However, after a few days, due to clinical deterioration of the patient, particularly progressive paraparesis, the multidisciplinary team planned a biopsy of the lumbar region.

After laminectomy of the third lumbar vertebrae, the dura mater was opened. However, no cerebrospinal fluid (CSF) flow was seen, and all nerve roots were edematous and dark gray in color. Samples were collected for biopsy from the posterior rootlets and arachnoid layer. The pathologic study of the samples revealed leptomeningeal melanoma with a densely cellular tumor, comprised of irregular masses of large pleomorphic cells and melanin in tumor cells. 

Numerous mitotic figures associated with nerve root invasion were detected (Fig. 3A-3B). However, immunohistochemical (IHC) staining was not performed with anti-MART-1, anti-melanosome, or anti-S-100 antibody. Dermatological and ophthalmological surveys showed no primary lesion in the skin or retina. The parents refused adjuvant therapy, and the patient died six months later.

**Fig-3 A&B:**
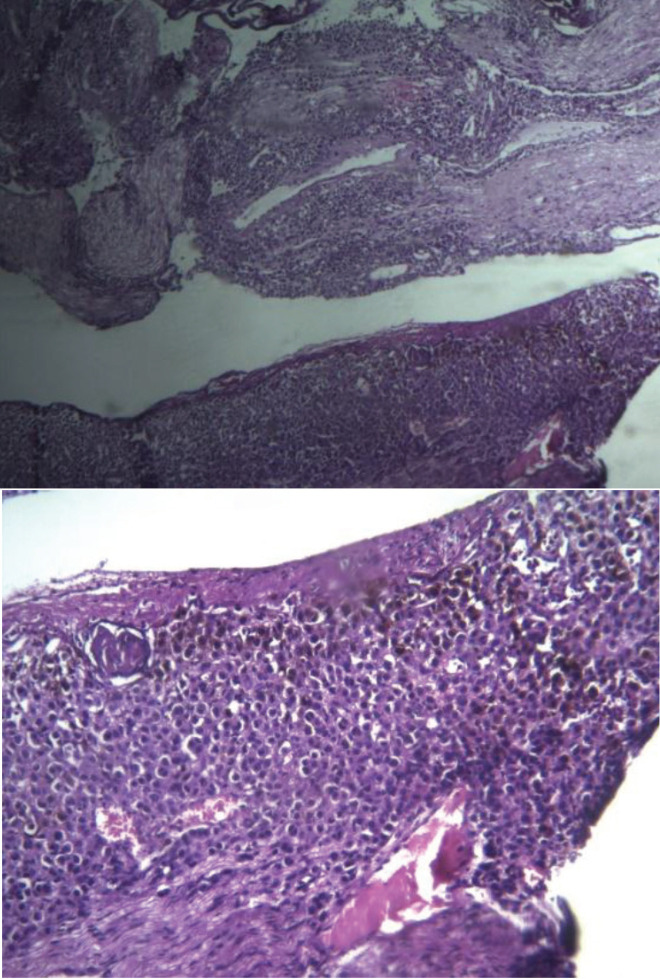
Hematoxylin-eosin-saffron staining ×200 (A) and ×400 ( B) showed tumoral cells containing melanin pigments and nerve root invasion

## Discussion

Primary diffuse leptomeningeal melanomatosis is a rare malignant melanoma of the CNS (4). Similar to other melanocytic neoplasms, it is caused by melanoblasts, which originate from the neural crest in early embryogenesis and move to the skin and other peripheral structures, including the leptomeninges (5). In the CNS, melanocytes are mainly found on the ventral aspect of the brainstem, particularly the medulla oblongata, acting as a detoxificator (6); however, the involvement of nerve roots may be also observed (7).

The symptoms of PDLM are nonspecific and are caused by intracranial hypertension and focal neurological deficits (1). Primary diffuse leptomeningeal melanomatosis may bear resemblance to different diseases, such as subacute meningitis, lymphoma, tuberculosis, leukemia, metastatic cancer, idiopathic hypertrophic cranial pachymeningitis, acute disseminated encephalomyelitis, and acute neurosarcoidosis (8, 9).

The typical MRI findings of PDLM include diffuse leptomeningeal hyperintensity on T1-weighted imaging, low T2 signal intensities arising from the paramagnetic features of melanin (10), hyperintensity on FLAIR images, and homogenous enhancement with gadolinium (11). However, the MRI findings in our patient were similar to amelanotic forms (5). These features in our case were completely nonspecific and could be associated with low melanin in the leptomeninges.

Cerebrospinal fluid analysis usually yields nonspecific results, indicating high levels of proteins, improved red blood cell count, normal (or low) level of glucose, leukocytosis, and occasionally characteristic pleomorphic neoplastic cells consisting of intracytoplasmic melanin pigments (12). In the present case, CSF sampling before surgery yielded inconclusive results, and intraoperative sampling provided nonspecific findings.

Immunohistochemical staining was not performed because of the typical finding on hematoxylin-eosin (H&E) staining for melanoma. To exclude extraneural sources (such as congenital syndromes), the implementation of different tests (including dermatological and ophthalmological tests) is necessary after definitive diagnosis (1, 3).

The efficacy of therapeutic interventions, such as corticosteroids, radiotherapy, chemotherapy, intrathecal interleukin-2, and surgery, is poor for hydrocephalus and only palliative (4, 5, 13, 14). 

## In Conclusion

Primary diffuse leptomeningeal melanomatosis is a rare and aggressive tumor with no specific presentations, posing challenges to its diagnosis and treatment. In case comorbidities are not definitively diagnosed in patients with unusual symptoms of meningitis, diagnostic methods such as CSF analysis and CNS biopsy can be helpful in identifying other underlying conditions.
